# Evaluating the Acceptability of a Brief Web-Based Alcohol Misuse Prevention Program Among US Military Cadets: Mixed Methods Formative Evaluation

**DOI:** 10.2196/67637

**Published:** 2025-04-16

**Authors:** Emily Schmied, Lauren Hurtado, W Ken Robinson, Cynthia M Simon-Arndt, Richard Moyer III, Leslie Wilson, Mark Reed, Shannon M Blakey, Marni Kan

**Affiliations:** 1 School of Public Health, San Diego State University, San Diego, CA, United States; 2 Institute for Behavioral and Community Health, San Diego, CA, United States; 3 Leidos Inc, San Diego, CA, United States; 4 Psychological Health and Readiness Department, Military Population Health Directorate, Naval Health Research Center, San Diego, CA, United States; 5 United States Air Force Academy, CO, United States; 6 San Diego State University Research Foundation, San Diego, CA, United States; 7 Counseling and Psychological Services, San Diego State University, San Diego, CA, United States; 8 RTI International, Research Triangle Park, NC, 27709, United States

**Keywords:** alcohol misuse prevention, digital interventions, formative research, military health, acceptability, alcohol prevention, US military, military, United States, formative evaluation, alcohol use, evidence-based prevention, alcohol intervention, mixed methods study, survey, alcohol use disorder, alcohol misuse, heavy drinker, educational web-based intervention, web-based intervention

## Abstract

**Background:**

As alcohol misuse remains pervasive within the military, evidence-based prevention programs that are feasible to implement and appropriately tailored to meet the needs and norms of military personnel are critically needed. Further, programs that target future military leaders, such as trainees, recruits, and cadets, may be especially impactful. eCHECKUP TO GO is a web-based, evidence-based brief alcohol intervention designed to reduce alcohol misuse through education and personalized feedback that may be suitable for military trainees. However, because it was developed for civilian students, efforts to adapt the content for military settings are needed.

**Objective:**

This study aimed to evaluate the acceptability of a military version of eCHECKUP TO GO, tailored to include military-specific terminology and alcohol use statistics.

**Methods:**

US Air Force Academy cadets were recruited to participate in a single-arm, mixed methods study. Following the completion of eCHECKUP TO GO, participants completed a survey that assessed satisfaction with specific aspects of the user experience, including ease of use, design, and relevance of the information and personalized feedback (range: 1=strongly disagree to 7=strongly agree). A subset of cadets also participated in a focus group to expound on the survey responses.

**Results:**

Survey participants included 22 cadets (n=12, 55% male; mean age 19.6, SD 1.8 years). In addition, 6 (27%) cadets participated in the focus group. Participants were satisfied with the program overall (mean 5.8, SD 0.9) and gave the highest ratings to ease of use (mean 6.6, SD 0.7), site design (mean 6.5, SD 0.6), and site interactivity (mean 6.4, SD 1.0). Items pertaining to tailoring, relevance, and amount of content specific to cadets scored lowest (mean 5.8, SD 1.4; mean 5.6, SD 1.4; and mean 5.5, SD 1.5, respectively). Most (n=15, 68%) participants said they would act upon the information they were provided. Focus group participants made suggestions for improved tailoring, such as increasing content on social aspects of drinking and military-specific risks of alcohol misuse (eg, Uniform Code of Military Justice violations).

**Conclusions:**

Although the acceptability of eCHECKUP TO GO was high, continued efforts are needed to ensure the content accurately reflects the experiences of cadets. Researchers who design military health promotion interventions need to consider the varied contexts within the force and rigorously evaluate the acceptability of all content before implementation.

## Introduction

Alcohol misuse is pervasive within the US military [1-4]. Data from the 2018 Department of Defense (DoD) Health Related Behaviors Survey indicated that binge drinking and heavy drinking [5] are highly prevalent (34% and 9.8%, respectively). For many service members, alcohol misuse may lead to serious adverse health, career, and social outcomes [6-8]. For instance, among all active duty service members, alcohol use disorder was among the top 10 causes of outpatient medical visits for both men and women in 2022 [2,3]. During the same period, alcohol-related issues were also a leading cause of inpatient hospitalization [3]. It has also been associated with earlier separation from service and disciplinary action [6,7]. Further, it has been noted that alcohol misuse during service may persist following separation [9,10], potentially contributing to the widely documented health disparities between veterans and civilians [11,12].

Alcohol misuse can pose serious threats to the health and performance of service members, thus interventions designed to prevent it before it causes lasting harm are urgently needed. Prior research has shown that the implementation of alcohol misuse prevention programs in the military is complicated by numerous factors, including the size, diversity, and wide geographic distribution of the force [11]. One possible approach is to target individuals in the earliest stages of their careers who likely already consume alcohol but have yet to develop problematic alcohol habits [13-15], namely those enrolled in military service academies or training settings. Another promising strategy is to use brief alcohol interventions, or BAIs, which often consist of singular sessions and thus require fewer resources to implement [16]. BAIs are endorsed for use among service members who screen positive for unhealthy alcohol use in the current Department of Veterans Affairs and DoD joint clinical practice guidelines for the Management of Substance Use Disorders [17].

BAIs have been used in military settings with varying results, though most reports are from studies conducted in veteran or general active duty populations [18]. The available evidence from training settings shows promise. For example, Klesges et al [19] evaluated the Alcohol Misconduct Prevention Program (AMPP) in Air Force technical training [20,21]. AMPP, which consisted of a combination of a single-session, group-administered BAI, and random breathalyzer tests, was associated with a significant reduction in alcohol-related risk incidents (eg, alcohol-related disciplinary infractions). While AMPP was found to be cost-effective [22], the continued implementation of in-person group trainings may not be sustainable in all settings. Further, because alcohol use is prohibited in military training settings [11], participant concerns about confidentiality may limit the impact of such methodologies [23]. Web-based BAIs that give personalized feedback to individual program participants may provide a solution to these challenges.

Prior web-based trainings (including BAIs) evaluated in active duty personnel and veterans have been shown to be acceptable and efficacious, but few have been evaluated in service academies or training settings, leaving a critical knowledge gap [24-27]. This study addresses this gap by assessing the acceptability of a web-based, individualized BAI among military cadets. eCHECKUP TO GO (alcohol-specific version; San Diego State University) is an evidence-based, theoretically driven web-based intervention originally developed for civilian adolescents and young adults. It is designed to reduce alcohol misuse by targeting cognitive risk factors (eg, peer normative behaviors) and increasing positive behavioral strategies (eg, increasing time between drinks) to minimize drinking-related risks by providing personalized feedback to participants. The efficacy of eCHECKUP TO GO has been repeatedly demonstrated in young adults [28-32]. Given the purpose, format, and prior success of eCHECKUP TO GO, it may be suitable for decreasing alcohol misuse in military academies. However, before implementing eCHECKUP TO GO, it is important to determine whether the format and existing content are acceptable among potential program participants. Specifically, while there are demographic similarities between civilian young adults and military cadets and midshipmen, the program may need to be tailored to account for cultural and contextual differences between the communities. The results of this study can be used to determine whether eCHECKUP TO GO may be appropriate for use in military academies or training settings.

## Methods

### Study Design and Procedures

Participants in this study were a convenience sample of 22 cadets enrolled at the US Air Force Academy (USAFA). Prospective participants were recruited through third-party announcements distributed by local study partners by email and word-of-mouth. Interested cadets attended a recruitment event where the study purpose and procedures were explained by members of the research team and those who provided written consent to participate immediately began their participation. A mixed methods design was used in which all consenting participants completed the eCHECKUP TO GO program (alcohol version) and an electronically administered, anonymous feedback survey. Immediately after completing the feedback survey, a subset of participants volunteered to participate in a 20-minute focus group discussion designed to expound on the content of the survey. The focus group was facilitated by two members (ES and LH) of the research team and was audio-recorded and transcribed for analysis. Data collection took place in January 2023.

### Description of Intervention

**Figure 1.**
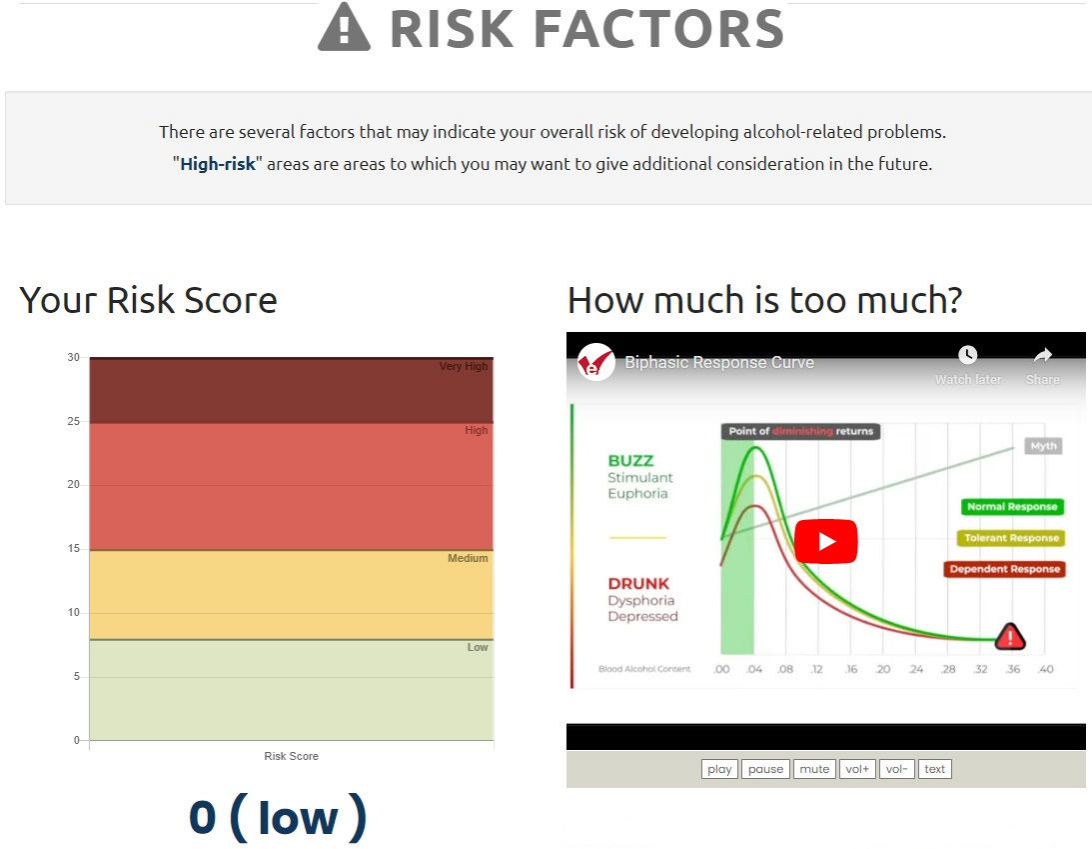
eCHECKUP TO GO example alcohol misuse risk feedback.

eCHECKUP TO GO is a brief, educational, web-based intervention designed to minimize alcohol misuse by providing personalized feedback. The program includes two components. First, participants complete a self-assessment, in which they are prompted to report their demographic characteristics (eg, age, gender, height, and weight), current alcohol consumption and behaviors (eg, frequency and quantity of drinking and driving while drinking), and alcohol-related cognitions (eg, reasons for drinking, such as “alcohol helps me reduce stress”). Participants also describe their goals and priorities, such as the importance of their fitness levels and relationships with others. The assessment also prompts participants to estimate the alcohol consumption behaviors of individuals in their peer groups (ie, people of the same age, gender, and occupation). Once the assessment is completed, participants are directed to a series of modules that provide personalized feedback based on the responses provided during the assessment. The feedback modules include a range of information. For instance, one module addresses perceived peer norms by showing how the participants’ alcohol consumption compares with that of their peers. Other modules show participants how much money they may spend on alcohol, their risk of becoming a problem drinker, and the potentially negative consequences of continued drinking based on their current use. Additional modules provide suggestions for how participants might change their habits and cognitions to reduce their risk for alcohol misuse and better achieve their self-reported goals (eg, alternate alcoholic drinks with water and set a drinking limit before going out). The version of eCHECKUP TO GO used in this study was adapted for use in military populations by incorporating service-specific terminology and imagery. Additionally, the peer normative feedback provided to participants was based on alcohol use data reported by Airmen in the most recent DoD Health Related Behaviors Survey [1], a trusted source used to inform military programming and resource allocation decisions. The program takes 20‐30 minutes to complete. Figures1  2 show examples of program feedback.

**Figure 2.**
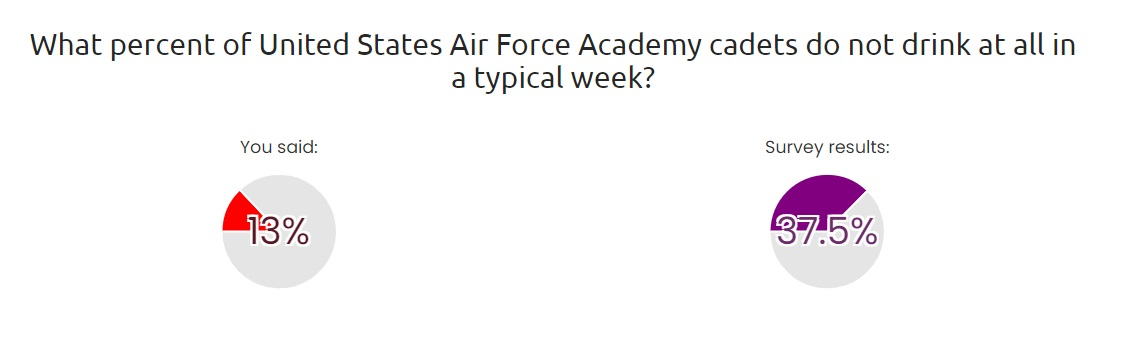
eCHECKUP TO GO example peer normative feedback.

### Ethical Considerations

All study procedures and measurement tools were reviewed and approved by the Institutional Review Board at San Diego State University (protocol HS-2022‐0009) and the USAFA Human Research Protection Program and Cadet Wing. Participants provided written consent to participate, and only those who did so were invited to complete the study activities. Survey data were collected without personal identifiers and only anonymized data were used for the study; all data were stored in a secured drive with access limited to approved study personnel. Participants received no compensation for participating.

### Measures

#### Survey

The self-report survey assessed participant demographic characteristics and satisfaction with various aspects of the eCHECKUP TO GO program, including the intended utility of the information. Sociodemographic characteristics included gender, ethnoracial identity, and age.

#### Satisfaction With eCHECKUP TO GO Content and User Experience

An abbreviated 11-item version of the general Web trust questionnaire [33-35] assessed perceptions of aspects of the program known to be important to individuals’ reviews of web-based health information: credibility of information (ie, “The advice seemed credible” and “I trusted the information on the site”), quality of the information and content (ie, “The site had a professional design”), and personalization of information (ie, “It felt like the advice was tailored to me personally”). Response options ranged from 1=strongly disagree to 7=strongly agree. Three original items designed by the researchers were added to obtain more specific information about participants’ perceptions of the program content. Two were included to assess satisfaction with the alcohol-related content (eg, “the feedback I received about alcohol use”), and another to assess satisfaction with the military content (ie, “amount of information about cadets or military service members”). Participants rated their degree of satisfaction on a scale from 1=completely dissatisfied to 7=completely satisfied. In addition to the rating scales, participants were asked to respond to two open-ended questions: “What did you like about the program?” and “How do you think the program could be improved to best help others?”

#### Use of Information

The intended outcome of eCHECKUP TO GO is to inspire behavioral changes related to alcohol, so participants were also asked at the conclusion of the program whether they intended to act on the advice they received related to alcohol consumption (yes or no). If yes, they were asked to explain in a free-response field which of the actions or strategies they intended to use, thereby providing an additional indicator of program satisfaction and the perceived relevance of the information [33].

#### Focus Group Guide

The focus group guide included nine questions that assessed participants’ perceptions of the eCHECKUP TO GO program. Participants were prompted to provide their opinions on the aesthetics, functional features, and content of the program (eg, “Tell me what you thought about the look and feel of the program” and “How relevant did you find the recommendations were to you?”). Participants were also asked for general feedback (ie, “What else can we do to improve the eCHECKUP TO GO program?”).

### Analysis

Quantitative survey data and qualitative data were first analyzed separately. Descriptive statistics, including means and frequencies, were computed for all close-ended survey items to assess the distribution and completeness of responses. Open-ended survey questions and focus group data were combined and analyzed using the rigorous and accelerated data reduction (RADaR) technique [36]. The RADaR technique was selected to provide expedited feedback about necessary program adaptations to be implemented before making eCHECKUP TO GO more widely available to cadets [37]. Using RADaR, two coauthors (ES and LH) engaged in an iterative process of data reduction and coding to identify pertinent themes related to the following study research questions: (1) What aspects of eCHECKUP TO GO were participants most satisfied with? and (2) How could eCHECKUP TO GO be improved for use among cadets? Once a codebook was finalized, two analysts (ES and LH) independently reviewed all study data and compared results to identify potential discrepancies. Few discrepancies were identified and all were resolved through discussion. Study analyses were completed by integrating quantitative and qualitative data to examine their convergence and complementarity, with results shown in a joint display [38,39]. Data collection and analyses were led by researchers without firsthand military service experience. The lead analyst (ES) is a former military beneficiary and the second analyst is a civilian (LH); both have experience conducting military health research.

## Results

### Overview

The survey participants were 22 cadets. A slight majority (n=12, 54%) were male. The mean age was 19.64 (SD 1.86; range 18‐26) years, with 6 (27%) participants aged 21 years or older. Approximately, 40% (n=9) of participants identified as non-Hispanic White, approximately one-fifth (n=4, 18%) of participants identified as Hispanic or Latinx or multiracial, 14% (n=3) of participants identified as Black or African American, and 4% (n=1) of participants identified as Asian Pacific Islander or Asian. When compared to the cadet population at large, this sample includes proportionally more female and individual participants from minoritized ethnoracial groups [40]. About one-quarter (n=6, 27%) also participated in the focus group. Demographic characteristics were not recorded for focus group participants to preserve their anonymity; thus, it is not possible to determine whether they significantly differed from the larger group. Further, participants were not asked to report their current or prior alcohol use behaviors because alcohol use is not permitted in this population or setting.

Results of the survey and focus group are jointly displayed in Table 1. Through analysis of the qualitative data, the research team identified three overarching themes addressed by participants: design and functionality, credibility of content, and tailoring and relevance of content. Further, it was observed that participant satisfaction with these aspects of eCHECKUP TO GO differed, with more positive responses provided about the design and functionality than the other themes. A review of the quantitative survey responses revealed a parallel pattern, such that items pertaining to design and functionality were rated highest, while items pertaining to tailoring and relevance of content were rated lowest. A summary of the feedback provided by participants is presented here by theme.

**Table 1.** Joint display of quantitative and qualitative responses demonstrating participant perception of the eCHECKUP TO GO program.

Theme	Survey responses and exemplifying quotations from open-ended survey items and focus groups
Design and functionality	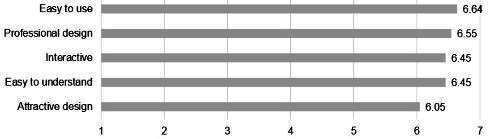 Exemplifying quotations: satisfaction “The program was very accessible and easy to understand in terms of direction.” [SP^a^ #19] “I liked how interactive the overall site was and how it gave me information based on my inputs*.”* [SP #1] Exemplifying quotations: suggestions for improvement “I think the program is overall very good. Some of the questions I misinterpreted so having like a small description below with a better or more explanation of the question could probably help.” [SP #7]
Credibility of content	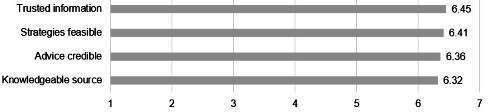 Exemplifying quotations: satisfaction “I appreciated the stats*.”* [SP #21] “I like that it gave a lot of helpful information on drinking and how to be a safer drinker.” [SP #16] “Yeah, I think it was very feasible. I guess, having the discipline to do it.” [FGP^b^ #3] Exemplifying quotations: suggestions for improvement “Provide more information and stats on alcohol use in the military, specifically cadets.” [SP #21]
Tailoring and relevance of content	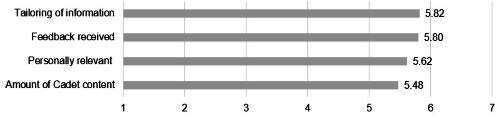 Exemplifying quotations: satisfaction “Yeah, I liked how if you didn’t put yourself down as drinking. It would, it was like, here’s things you can do to recommend to others instead of you need to do these things.” [FGP #2] “I think by asking us to input what we thought the statistics were and what was important to us made it much more personal.” [SP #12] Exemplifying quotations: Suggestions for improvement “You had all this stuff like with the airmen and cadets, but when you went to the consequences, it was largely just like social and physical consequences, maybe like help emphasize like the military aspect of it ... Just to reinforce that like drinking can ruin your career, which is what we’re all here to set up for.” [FGP #2] “Provide more military-tailored advice ([it’s a] different drinking culture here than civilian schools).” [SP #10]

a SP: survey participant.

b FGP: focus group participant.

### Design and Functionality

On the survey, participants were most satisfied with the ease of use (on the 1‐7 scale: mean 6.64, SD 0.73), how easy it was to understand the information (mean 6.45, SD 0.91), and the design and interactivity (mean 6.55, SD 0.60; and mean 6.45, SD 1.01, respectively). The qualitative data additionally elucidated the design features of the program most liked by participants. Several survey respondents commented on the features of the program that made it easy to use. For example, one participant wrote, “I thought it was clean and easy to follow and did not have unnecessary filler” [survey participant (SP) #17]. In the focus group, a participant who appreciated the embedded skip logic that allowed participants to advance throughout the assessment based on their responses said, “I liked in the parts where it was like kind of the yes or no that would just automatically move down to the next question instead of like having to scroll after answering each one” [focus group participant (FGP) #4]. Others commented on the interactivity of the program (“I liked how interactive the overall site was and how it gave me information based on my inputs” [SP #3]).

When it came to suggesting improvements in the design and functionality, most participants commented on the self-assessment component of the program. Several participants described difficulty completing the drinking history questions, which are structured in the format of a calendar where respondents enter the number and type of drinks they consumed on different days of the week and during certain hours. In the focus group, two participants described difficulty with the calendar, stating that “the calendar was too stressful” [FGP #2] and “I don’t pay attention to, like, how many drinks I had an hour or in the specific hours, which like, how long I’ve been drinking” [FGP #3].

### Credibility of Content

Survey responses revealed a high degree of trust in the information and suggestions provided in eCHECKUP TO GO, as indicated by the high scores for credibility of information (mean 6.36, SD 0.66) and feasibility of alcohol misuse prevention strategies provided (mean 6.41, SD 0.79). The qualitative results helped to explain the highest ratings, as the participant responses revealed an appreciation for data, which are frequently presented to eCHECKUP TO GO users. One respondent noted, “I appreciated the stats” [SP #21], while others remarked, “I liked how it showed graphs and other ways for people to see how those products affect themselves and their peers” [SP #4], and “[I liked the] statistics at the end, as well as the estimation questions to display knowledge” [SP #10]. Others noted the specific information they recalled, such as “I really liked the part where it told you about like the calories and like how it can impact muscle mass, especially because physicality is such a big thing here that if, like, if people are drinking, they just don’t realize the calories and how it can hurt you in your muscles” [FGP #2]. Further showing the importance of data to participants, the suggestions provided related to the credibility of content were to add even more data (“Maybe more data and statistics” [SP #12]).

### Tailoring and Relevance of Content

As shown in Table 1, the scores for tailoring and relevance of eCHECKUP TO GO content were relatively lower (ie, amount of information about cadets: mean 5.48, SD 1.57). Qualitative responses provided context for these lower scores, showing that while many participants appreciated the personalized feedback they received, they felt that the content did not include enough information tailored to the unique experiences of military cadets. In terms of the personalized feedback, one participant noted that what they liked best about the program was it was “very personally [sic] to me, showed me the statistics, how much I am at risk” [SP #22], while another said, “I think personally it made me more aware of how much I was drinking or how much others are drinking ... it can make us more aware of our habits and our spending” [FGP #3].

When describing opportunities to improve the program tailoring, focus group participants discussed a need for more information on the social aspect of drinking. One participant explained:

 *There could be ... a lengthy section for the social aspect of drinking ... a lot of cadets just go out and drink together. And I don’t think the survey emphasized that really enough. I think it was individually tailored, which I think is good. But also, it didn’t go into the, too much, in my opinion, to the causes of like why people go out and drink, maybe just because of like the social pressure, because again, especially being cadets, there’s always that implied social pressure*.

[FGP #2]

Another participant also suggested adding more content on peer pressure and drinking culture, saying, “I feel like there should be a section on ... how much of the overall culture, overall climate of drinking contribute to your own and like do others pressure you to drink and stuff like that” [FGP #4]. Relatedly, another cadet requested content about “how to help friends and notice if they have an alcohol problem” [SP #22].

Participants recommended improving content tailoring to address other aspects of cadet or military life as well. Some felt that the existing content of eCHECKUP TO GO did not consider the unique schedules of cadets. They explained that there are periods of time when they are forced to completely abstain from drinking and do not have access to alcohol and then periods where it is almost expected that they would indulge, making it hard to answer the self-assessment items that asked about behaviors in the past week or a “typical” month. According to one participant, “We don’t get off base much. So, our drinking habits may look very irrational, making it hard to say I drink x on a typical week” [SP #16]. Other suggestions pertained to the personalized feedback provided about the consequences of drinking. It was suggested that the military-specific consequences be included, which previously were not in the program:

 *When you went to the consequences, it was largely just like social and physical consequences. Maybe, like help emphasize, like, the military aspect of it*. [Such as] *like this many people a year get put on alcohol probation here* [or] *like in the actual Air Force, like this many people get in trouble for drinking, and like drinking can lead to these Uniform Code of Military Justice violations and has been linked to them or something like that. Just to reinforce that like drinking can ruin your career, which is what we’re all here to set up for*.

[FGP #2]

Another noted that the eCHECKUP TO GO program provided feedback about how much money they spent on alcohol compared with a rent payment, which cadets do not pay, adding, “So, we don’t pay rent or at least we don’t see it ... So, I guess if it’s specific to cadets, maybe just don’t add that” [FGP #3]. Finally, some requested “more military-tailored advice (different drinking culture here than civilian schools)” [SP #21].

### Future Use of Alcohol Misuse Prevention Strategies

Over two-thirds of participants (n=15, 68%) said they intended to use at least one alcohol misuse prevention strategy they learned from eCHECKUP TO GO. Those who answered affirmatively were asked to describe which, if any, of the alcohol misuse prevention strategies learned from eCHECKUP TO GO they would use in the future. Through qualitative analysis, it was determined that responses could be categorized into two salient themes: strategies to reduce the amount consumed and strategies to protect others. Several participants planned to reduce their intake by spacing out their drinks, saying, “I intend to use the ‘spacing drinks over time’ strategy” [SP #3] and “When reaching the legal drinking age, to practice safe drinking habits like spacing out drinks and setting limits for myself” [SP #20]. Additionally, numerous participants planned to use strategies to protect their peers, such as one participant who explained, “Even if I am not drinking and don’t have to worry about putting myself in danger, I can look out for my friends when they are drinking and make sure they are making good decisions regarding alcohol” [SP #1]. Others indicated that they would act as the designated driver for their friends, stating, “I will likely be the DD for my group on more occasions” [SP #17].

## Discussion

### Principal Findings

Addressing the longstanding issue of alcohol misuse in the military requires creative solutions designed to mitigate the challenges inherent to promoting health in large, diverse communities [1,2,4,11,41]. This study sought to determine whether a military-tailored version of eCHECKUP TO GO is an acceptable option for use among military cadets, recruits, or trainees. Study results identified aspects of the program that were well received by participants, including the web-based format and interface, and the personalized feedback about alcohol risk and protective strategies. Importantly, no concerns were expressed by participants about confidentiality. These findings suggest that this internet-based, individually completed BAI may be acceptable in this community, providing a possible solution to the logistical issues and privacy concerns that often impede the implementation and efficacy of health promotion programs in military settings.

Although eCHECKUP TO GO was largely endorsed by participants, opportunities for improvement were identified. Though participants liked the self-assessment and personalized feedback, many felt that these modules did not sufficiently acknowledge the broader social context impacting alcohol use, particularly cultural norms and peer influence. Heavy alcohol use is widely known to be a norm within the military and among young adults, and research has shown that consuming alcohol with peers may be viewed as an opportunity to build cohesion [41-46]. Participants touched on these influences when they described situations in which they felt expected to drink or chose to drink to avoid feeling left out. These findings align with a study by Meadows et al [43] that found positive associations between perceived alcohol culture within respondents’ command or unit and binge and heavy drinking. While alcohol use is an individual behavior, it cannot be disentangled from the social context in which it occurs when working with military cadets or trainees [43]. Thus, future alcohol misuse prevention interventions may be more acceptable and efficacious if they directly address the impact of peer influence and provide concrete strategies for navigating social situations in which alcohol may be present.

Study results also highlight another cultural norm that should be incorporated into eCHECKUP TO GO and other alcohol misuse prevention programming: service before self. “Service before self” is a core value of the Air Force [47], and the other service branches uphold similar maxims. Feedback from study participants indicates that their ingrained commitment to service should be used as a cornerstone of alcohol misuse prevention content. For example, participants who intended to act upon the advice received as part of the program largely intended to use strategies to protect their comrades, and some asked for more content on how to help recognize alcohol misuse in others. Further, participants felt the program could be more impactful if it included information about career consequences of alcohol misuse, such as the Uniform Code of Military Justice violations. These findings parallel results from a study by Aycock et al [48], which also noted that alcohol misuse prevention messaging for Airmen should emphasize adverse career impacts. Framing alcohol misuse interventions as a means of protecting comrades and fulfilling a commitment to serve could bolster buy-in and work to dismantle longstanding alcohol-related cultural norms [41-43].

### Limitations

Methodological limitations must be considered when interpreting the results of this study. Due to the relatively small sample size and unique population, the results may not generalize to the USAFA community or other military service academies. Additional formative research may be needed to finalize intervention refinement before subsequent efficacy trials. In particular, more data may be needed to ensure the experiences and preferences of participants from minoritized communities who have historically been underrepresented in research are considered. Additionally, while no concerns about anonymity were expressed by participants, it remains possible that participants did not feel comfortable discussing their opinions about alcohol use out of fear of accidental disclosure of their identity. However, the responses received likely reflect participants’ opinions and behaviors because participants were given the option of skipping questions they did not wish to answer. Finally, as this was a formative evaluation, the results cannot be used to estimate the efficacy of eCHECKUP TO GO completion on alcohol misuse among cadets. The next steps in this line of research should include an efficacy trial powered sufficiently to determine the impact of eCHECKUP TO GO completion on attitudes toward alcohol misuse, alcohol-related protective behavioral strategies, and alcohol consumption among military cadets.

### Conclusions

The results of this study indicate that a military version of eCHECKUP TO GO may be suitable for use in military training settings, though additional tailoring is needed to account for the unique norms and values of this community. Study findings have important implications for the development and implementation of military alcohol misuse prevention programs, but the lessons learned can also be applied to programs that address other health behaviors. In communities that value service above self, program content should emphasize the ways in which improving individual health behaviors can benefit the community at large.

## Abbreviations

AMPP: Alcohol Misconduct Prevention Program
BAI: brief alcohol intervention
DoD: Department of Defense
FGP: focus group participant
RADaR: rigorous and accelerated data reduction
SP: survey participant
USAFA: US Air Force Academy
